# Home Care Technology Through an Ability Expectation Lens

**DOI:** 10.2196/jmir.3135

**Published:** 2014-06-20

**Authors:** Gregor Wolbring, Bonnie Lashewicz

**Affiliations:** ^1^Community Rehabilitation and Disability StudiesCommunity Health SciencesUniversity of CalgaryCalgary, ABCanada

**Keywords:** ability expectation, ableism, home care, home care technology, health care technology, people with disabilities, sensor devices and platforms, social robotics, health information gathering, participatory design

## Abstract

Home care is on the rise, and its delivery is increasingly reliant on an expanding variety of health technologies ranging from computers to telephone “health apps” to social robots. These technologies are most often predicated on expectations that people in their homes (1) can actively interact with these technologies and (2) are willing to submit to the action of the technology in their home. Our purpose is to use an “ability expectations” lens to bring together, and provide some synthesis of, the types of utility and disadvantages that can arise for people with disabilities in relation to home care technology development and use. We searched the academic databases Scopus, Web of Science, EBSCO ALL, IEEE Xplore, and Compendex to collect articles that had the term “home care technology” in the abstract or as a topic (in the case of Web of Science). We also used our background knowledge and related academic literature pertaining to self-diagnosis, health monitoring, companionship, health information gathering, and care. We examined background articles and articles collected through our home care technology search in terms of ability expectations assumed in the presentation of home care technologies, or discussed in relation to home care technologies. While advances in health care support are made possible through emerging technologies, we urge critical examination of such technologies in terms of implications for the rights and dignity of people with diverse abilities. Specifically, we see potential for technologies to result in new forms of exclusion and powerlessness. Ableism influences choices made by funders, policy makers, and the public in the development and use of home health technologies and impacts how people with disabilities are served and how useful health support technologies will be for them. We urge continued critical examination of technology development and use according to ability expectations, and we recommend increasing incorporation of participatory design processes to counteract potential for health support technology to render people with disabilities technologically excluded and powerless.

##  Introduction

Home care, consisting of provision of health support and resources within a person’s private residence, is an increasingly preferred model of care for older people and people with disabilities who are in constant need of some form of health intervention [1-5]. Health technologies continually reshape what is possible in home care and the creation of home environments that support health clients. Further, a health care discourse is emerging from expectations for health clients to be in control of their health interventions (see concepts of patient-driven health care and people-driven health research) [6]. We see movements towards a “quantified self” (where people diagnose themselves), health social networks, and participatory medicine with an active health science and technology market that makes consumer personalized medicine possible [6]. Furthermore, people increasingly seek health information by themselves. These developments transform the very meaning of health, health care and rehabilitation [7,8], and the delivery of health care and rehabilitation [9-16] including home care. This in turn changes expectations for the functional abilities of those considered suited for home care support. This shift in understanding of the nature of the health client from a passive recipient to an active shaper, and a corresponding framing of patients and clients as health consumers, has broad implications for people with disabilities.

In this paper, we focus on ways that health technologies may change ability expectations for people receiving home care. We critically examine how technology-driven products and processes, including those related to self-diagnosis, health monitoring, companionship, health information gathering, and care, pose new challenges in terms of physical and cognitive accessibility for people with disabilities. Non-normative body abilities present challenges for physically accessing health technologies while non-normative intellectual abilities raise obstacles for understanding information provided via health technologies and for understanding how to operate a given health technology. In this paper, we use the lens of ability expectations (that one wishes to possess certain abilities and regards these abilities in others as desirable) and ableism (where such ability expectations are viewed as not only desirable but also essential) to examine the impact of ability expectations inherent to using self-diagnosis, health-monitoring, and care devices. In short, this lens is used to focus on what abilities are expected and why, and the impact of such ability expectations [17,18]. We anchor this work in the belief that ableism is an important consideration in how ableist thinking influences the development and use of health technologies and ultimately the direction of advances in health care, health research, health policy, and the utility of these for people with disabilities.

## An Ability Expectation/Ableist Lens Described

The term ableism evolved from the disabled people rights movements in the United States and Great Britain during the 1960s and 1970s [19]. It has been traditionally understood in the disabled people rights movement and in the academic field of disability studies as a grand narrative, that is, a universalized and systematized conception of “ability” oppression based on a set of beliefs, processes, and practices that perceive species-typical normative body structure-based abilities as essential. Ableism was coined to flag that certain physical and mental abilities were not only desired but also viewed as essential. Ableism can lead to the experience of disablism [20], which means a lack of equity for people who do not exhibit expected abilities and are thus labelled as sub-species-typical, namely people labeled as having disabilities. Yet ability expectations and ableism are much broader cultural phenomena not confined to physical and mental abilities. Every individual, household, community, group, sector, region, country, and culture cherishes and promotes numerous abilities while viewing other abilities as non-essential or even undesirable. Many people desire the ability to consume certain products, be competitive, or productive while others desire living in an equitable community. Some desire the ability to drive a car, others the ability to use public transportation. The pervasiveness and infinite possibilities of ability expectations produce a particular understanding of oneself, one’s body, and one’s relationship with others of one’s species, other species [21], and one’s environment [21] based on one’s abilities [17,18]. A self-perpetuating pattern plays itself out through which ability expectations shape health technology development and use. In turn, ability expectations inherent to available health technologies influence the ability expectations of people with disabilities and whether those people can fulfil the ability expectations becomes a defining factor of the utility of such health technology products for people with disabilities.

This paper is our attempt to momentarily apprehend this ever-evolving pattern to draw out the utility and potential disadvantages so-called supportive technologies may pose for people with disabilities. We approach this through a critical examination of a selection of academic literature. We collected articles from the academic databases Scopus (n=72), Web of Science (n=19), EBSCO ALL (n=27), IEEE Xplore (n=6), and Compendex (n=6) that had the term “home care technology” in the abstract or as a topic (in the case of Web of Science) (May 10, 2014). We also used our background knowledge and related academic literature pertaining to self-diagnosis, health monitoring, companionship, health information gathering, and care that we have used in earlier work. We examined these articles in terms of ability expectations assumed in the presentation of home care technologies, or discussed in relation to home care technologies. Our purpose is to bring together, and provide some synthesis of, the types of utility and disadvantages that can arise for people with disabilities in home care technology development and use.

## Living in an Age of Health Support From the Convenience of Home

### Health Information Gathering

As part of a widespread “do-it-yourself” move towards obtaining health information from online sources without involving a health practitioner, health clients increasingly expect to be in the driver’s seat with their health interventions [22]. Ideas of *patient-driven health care* have become central in policy discussions as quantified self-movements continue to gather momentum predicated on people’s ability to diagnose themselves [6,10-13]. A 2006 survey of more than 8000 Americans responding to an offer of free Internet access in exchange for completing occasional surveys, yielded findings that “populations with serious health needs and those facing significant barriers in accessing health care in traditional settings turn to the Internet for health information” [23]. Indeed, people increasingly self-diagnose aspects of their “health” [12]. According to the Wisconsin Longitudinal Study Graduate Sample, 40% of respondents with Internet access reported using the Internet to look for advice or information about health or health care in 2001 [24]. Findings from a US study show that 80% of American Internet users have searched for information on at least one of 17 health topics [25]. According to USA Today, search engines such as AOL and Google (whose top 10 list of most frequently visited sites for 2009 overlaps somewhat with AOL’s) are typically the first place Internet health information seekers visit, rather than academic medical center or hospital websites [26]. The Pew Research Center’s Internet & American Life Project, which consists of ongoing surveys about the social impact of the Internet, concurs that in 2008, “8 in 10 Internet users, or 61% of US adults, have looked online for health information” [27]. In fact, 60% of respondents to that survey indicated the information found online affected a decision about how to treat an illness or condition while 56% say it changed their overall approach to maintaining their health or the health of someone for whom they provide care [27].

### Health Monitoring and Care at Home: Sensors, Assistive Devices, and Social Robotics

The Ontario Homecare Association [28] highlights the responsive and fiscally beneficial nature of home care:

Home care has evolved by responding to changes that have occurred in the hospital sector (bed closures, increase in ambulatory care clinics, and day surgery) and in the long term care facilities sector (waiting lists for beds, limited availability). As a result, home care has emerged as an integral component of Canada’s health care system and essential to its sustainability. Home and community care comprises 4.25% of the overall spending on health care within provincial budgets

Health technology developments expand the ways in which the home is part of health care delivery. Sensor networks and ubiquitous computing are two leading technology developments that enable what is being termed “smart home care”. Smart home care entails, among other things, the use of miniature sensors and is designed to assist the elderly and chronic patient in ways that “integrate with existing medical practices and technology” and “enable real-time, long-term, remote monitoring” [29]. Sensors can be implanted into the body, externally attached to bodies (wearable sensors), and/or positioned in the walls and floors of a home [6]. Sensors are used for disabled people in areas such as health monitoring (eg, physiological monitoring such as through using sensor pillow systems to monitor for cardiorespiratory and posture movements during sleep, monitoring of movement, and detection of falls during waking hours) and provision of information (eg, assistance with indoor navigation or evacuation and rescue instructions in case of emergencies) [6].

Interactive devices also occupy an expanding place in home health monitoring and care. For example, wireless personal digital assistants (PDA) for telemedical diabetes care enable communication between a glucometer, an insulin pump, and a continuous glucose sensor controlled through the patient’s PDA device and responded to by the patient through a user-friendly interface [30]. Expanding on home health support possibilities is the newly emerging field of social robotics [31]. Social robots are designed to perform functions previously performed by health care staff ranging from monitoring nutrition and hydration and providing reminders to take medications to performing household tasks such as cleaning to highly interactive functions such as providing assistance with movement, providing companionship, and even offering motivational advice for physical activities [31].

### An Ability Expectations Critique

#### Overview

While we applaud advances in health care support made possible through emerging technologies, we urge critical examination of such technologies and their implications for the rights and dignity of people with diverse abilities. Using an ability expectation lens to examine technologies, we note concerns with potential for technologies to result in new forms of exclusion and powerlessness.

#### Exclusion

At first blush, the pervasiveness of accessing health information from online sources appears to be an important step towards a democratization of health information. Yet ease of access is far from democratic. For example, 3000 randomly sampled adults (2006) expressed frustration with a lack of information or an inability to find what they were looking for online, while 18% indicated feeling confused by information they found online [25]. These results may be unsurprising given that health information is not often presented in plain language and contradictory claims about health conditions are not uncommon. At the same time, the online or telephone survey designs of these large scale studies imply that respondents were of cognitively normative functional ability as a prerequisite for participation. If a significant proportion of these study respondents express frustration or confusion in relation to Internet health information, how might people labelled cognitively impaired be able to generate and evaluate Internet health information? Of related concern are ability issues for people with sensory differences (eg, blindness). Most webpages are not accessible to people who are blind or partially sighted [32]. Thus difficult questions arise over how people with sensory differences might be excluded from accessing information about their health.

Additional layers of exclusion arise given potential for use of sensors or robots by people with disabilities to result in decreased human interaction owing to sensors or robots replacing hands on/relationship-based health care providers [6]. Tiwari et al (2010) take concerns over reduced human interaction a step further as they speak about diminished interactions not with paid health providers, but rather with family caregivers. Specifically, Tiwari et al raise the potential for use of robots to give “permission” for family caregivers to abdicate responsibility in the lives of people with impairments (in this case, frail elders) on the pretext that their elders have artificial company [33].

#### Powerlessness

Health technologies raise a host of potential for people with disabilities to experience powerlessness through restrictions of access to, and control over, devices, along with restrictions in the very processes of consenting/approving use of devices.

Many, if not all, health technologies that expect “patient” interaction, such as the telemedical diabetes care devices, raise questions about “user friendliness” given that procedures requiring patients to generate, interpret, and relay information may not be accessible for people with physical or cognitive differences. Similarly, some functions of social robots require cognitive, physical, and in some cases, emotional abilities from the disabled person in order for the social robot to be of use.

Other monitoring and support features of devices and social robots may be viewed as “ability neutral” in terms of interaction expectations placed on the person being monitored, that is, the person is not required to program, interpret, or actively respond. However, these devices and robots raise ethical concerns pertaining to the ability of people with impairments to fully understand use of the devices, which in turn raises potential for invasions of privacy [6,24]. And what about instances where people may understand but not agree to terms of use? Tiwari et al (2010) discuss potential dilemmas over when and how individuals control their use of technology: “The ethical dilemma arises as to when to give a choice, where a user may allow or block certain features, eg, a user should have choice when not to allow observational recording but it also may be a compromise on safety when a potentially lifesaving device was turned off and it was needed” [33]. Indeed, several authors covering home care technologies mention the lack of ethical considerations [33,34], and one study highlighted that there are various ethical issues that have to be overcome and that ethics issues are very complex and subject to change as technology advances [35].

## Discussion

### The Need for Participatory Design Processes

Although the relative abundance and accessibility of prescribed health monitoring and support devices and Internet-based health information have expanded the reach of health support in general, accessibility and utilization for people with physical, cognitive, or sensory differences have not been accounted for in the design of many such health technologies. We argue that physical and social realities of people with disabilities command greater attention toward understanding and increasing accessibility of health technologies. People with disabilities are likely to experience more intense and complex health needs as they have relatively less access to social determinants of health such as economic security, social inclusion, and access to health promotion [36]. Further, adults with disabilities are not likely to have spouses and children to turn to for support [37,38]. While this population has high needs for access to health support, the technology advancements in health support discussed above may constitute a further layer of health support disadvantage owing to ableist attitudes and policies and practices flowing from such ableist attitudes. Through bringing together and synthesizing the types of disadvantages that can arise for people with disabilities in home care technology development and use, we delineate these disadvantages as falling into two main categories of exclusion and powerlessness. We conclude with resources and recommendations for taking steps to address these disadvantages.

Consistent with the principles indicated in research guidelines, which require collaboration with patients/participants [39], we suggest that problems of technology-driven exclusion can be addressed by product development that uses participatory design principles [40,41], where co-designing with generative design tools is one possible avenue to perform participatory design [42,43].

At the same time, a number of researchers point out difficulties associated with participation by people with disabilities [39,44,45] including needs for adapting processes through which informed consent, as well as study-topic related needs and preferences, are provided by participants. We refer readers to resources created to facilitate such adapted processes including Alberta Human Services, Adult Guardianship and Trusteeship Act (AGTA), which details guidelines for supporting adults who need assistance with decision making, and the Law Commission of Ontario, which provides a recent (2014) collection of commissioned papers pertaining to capacity, decision-making, and guardianship.

Rice et al (2007) discuss elder-friendly technology development and offer strategies for addressing an array of issues related to anxiety with technology, needs for concrete examples, and reluctance to complain [46]. Goodman et al also discuss difficulties engaging elders in product design and propose a model of “development by proxy” instructive to participatory work with people with disabilities [47]. Goodmann et al discuss the Prosumer (producer+consumer) Model of participatory design of technology for home health, which is based on a user-centered design process consisting of user needs assessment, technology prototype deployment to the home, in-home usability testing, feedback, and iterative design [47]. Prosumers with severe physical disabilities are trained in techniques of introspection and self-reporting to assist them to participate in usability studies. Their residences are being equipped with Internet-connected PCs that are smart-home interfaces with control and data logging software [47].

### Conclusions

We have provided a glimpse of what is possible, and for whom, in home self-diagnosis, health monitoring, companionship, health information gathering, and care. Ableism influences choices made by funders, policy makers, and the public in the development and use of home health technologies and impacts how people with disabilities are served and how useful health support technologies will be for them. We urge continued critical examination of technology development and use according to ability expectations, and we recommend increasing incorporation of participatory design processes to counteract potential for health support technology to render people with disabilities technologically excluded and powerless.
